# Supplementary figures and images for: PIWI silencing mechanism involving the retrotransposon nimbus orchestrates resistance to infection with Schistosoma mansoni in the snail vector, Biomphalaria glabrata
Source: PLoS Negl Trop Dis. 2021 Sep 8;15(9):e0009094. doi: 10.1371/journal.pntd.0009094 (PMC8462715; doi:10.1371/journal.pntd.0009094)

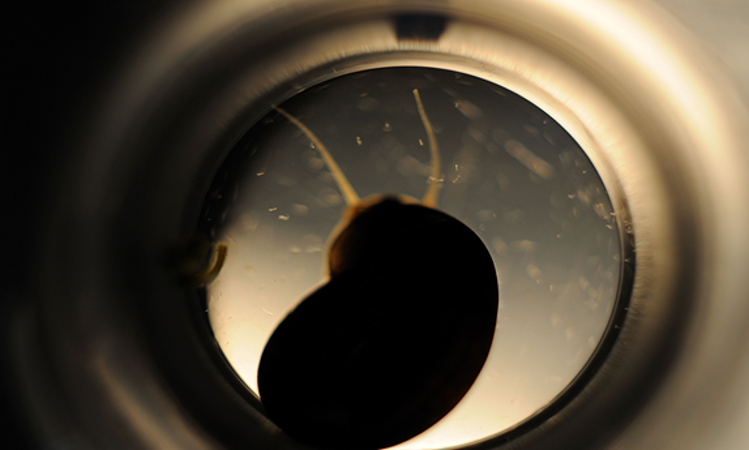

Supplement: S2 Fig — To determine the biological effect of silencing BgPiwi in relation to S. mansoni infection in BgPiwi siRNA/PEI transfected BS-90 snails, schistosome exposed BgPiwi siRNA transfected and untransfected snails were left at room temperature and evaluated at 4- and 6-weeks post-exposure. Note that the BS-90 snail transfected with BgPiwi siRNA shed cercariae at 4 weeks post-exposure to S. mansoni. (TIF) [file pntd.0009094.s002.tif]

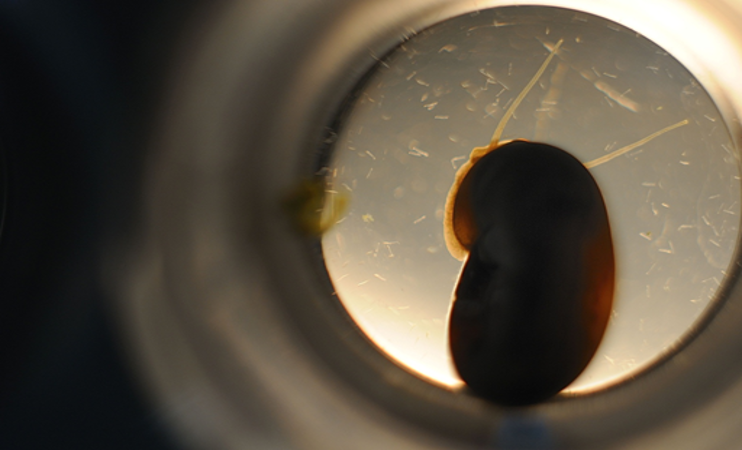

Supplement: S3 Fig — To determine the biological effect of silencing BgPiwi in relation to S. mansoni infection in BgPiwi siRNA/PEI transfected BS-90 snails, schistosome exposed BgPiwi siRNA transfected and untransfected snails were left at room temperature and evaluated at 4- and 6-weeks post-exposure. Note that the BS-90 snail transfected with BgPiwi siRNA shed cercariae at 6 weeks post-exposure to S. mansoni. (TIF) [file pntd.0009094.s003.tif]
