# Supplementary material for: PIWI silencing mechanism involving the retrotransposon nimbus orchestrates resistance to infection with Schistosoma mansoni in the snail vector, Biomphalaria glabrata
Source: PLoS Negl Trop Dis. 2021 Sep 8;15(9):e0009094. doi: 10.1371/journal.pntd.0009094 (PMC8462715; doi:10.1371/journal.pntd.0009094)
Supplement: Attachment — Submitted filename: review.docx [file pntd.0009094.s004.docx]

This manuscript describes the findings of a comprehensive study on the interplay of piwi and nimbus, and their potential role in the resistance or susceptibility of *Biomphalaria* snails to schistosome infection. This research is original and important to the field. It will also be of interest to researchers in other fields such as epigenetics and invertebrate immunology. Overall I recommend the manuscript be accepted following major editing. I recommend the following points be addressed and I’ve listed suggested edits.

- Overall, there was variation in formatting and writing style, perhaps as there are multiple authors. I recommend revising the paper so it is more uniform.
- It would be helpful to clarify why the 2 different siRNAs were used together rather than separately.
- Can the authors provide any explanation for why in Fig 3, UNI siRNA treated unexposed BS-90 showed a much greater decrease in nimbus expression than in UNIsiRNA treated and schistosome-exposed BS-90?
- This was a comprehensive study comprising several different methods/experiments. A flow chart or graphic giving overview of all methods would be helpful to readers.
- Likewise, a graphic summarizing the results would be helpful.
- The description of methods used to acquire sequences in order to design primers for qPCR were confusing (lines 209-223). There was mention of exploring the phylogeny of the *B. glabrata* orthologs but it isn’t clear why this was performed and I didn’t see the resulting data anywhere.
- If space in journal is limited, Figures 2B and 2C do not appear to be absolutely necessary. A table with number cercariae shed might be more impactful.
- The significance of the use of the BPPA hTERT inhibitor, and that it stops cercarial shedding when snails treated with it 2 weeks post-infection, was not clear.
- It was not clear why the 2 siRNAs were used simultaneously not individually.

**Suggested edits**

Line 37-38 – swap permissive and non-permissive

Line 42 – join acetyl and transferase = acetyltransferase

Line 42 – need to separate histone acetyl transferase and histone deacetylase with comma

Line 139 – suggest changing “obtain leads” to “obtain candidates” to avoid having leads twice in same sentence

Line 194 – insert comma after “(TmHMM)

Line 203 – please include more detail on how specific transcripts to be examined vi two step qPCR were selected.

Line 209 – not clear what “#7050” refers to. Should this citation be a number in refs rather than name and year?

Line 216 - join acetyl and transferase = acetyltransferase, insert comma after (*BgHAT*)

Line 228 – join “Bio” and “systems”. Is Wolston Warrington the correct address? I could not find it on a map. Maybe just include Warrington

Lines 208-226 – there is lack of consistency in wording of two step RT qPCR (i.e. sometimes two step included other times not), and whether given in full or abbreviated

Lines 244-75 – P of *BgPiwi* was uppercase previously

Line 246 – would change wording to “either dsRNA or siRNA, complexed with PEI”

Line 274 – remove “in”

Line 286 – have already given Sigma address in full (line 252)

Line 286 – insert comma after “MO)”

Line 310 – should the comma be replaced with a semicolon?

Line 317 & 328 – perhaps change “presented” to added

Line 330-1 – would reword description of incubations at different temperatures, e.g., The Top Brite was set for 2 min at 37C, then 2 mins at 75C, and then 30 min at 37C

Line 352 – numbers can be in same box, don’t’ need separate box for each number

Line 366 – De-novo doesn’t need uppercase d or a hyphen

Line 368 – should be one sentence, no period after “temperatures”

Line 395 – “demonstrates” rather than “demonstrate”

Line 399, “he” should be “the”

Line 426 – insert comma after “*nimbus*”

Line 428 – after “PEI)” insert a comma and then “ the”

Line 456 – remove “either”

Line 509 – insert space after “insects”

Line 514-19 – insert some commas in lists of genes, overall this sentence doesn’t read well

Line 528 – insert commas in list of transcripts (e.g. after (HAT) and after (HDAC)

Line 546 – replace “preformed” with “examined” or “recorded”

Line 551 – delete “reveals the repertoire”

Line 592 – can put 2 numbers in same [ ]

**Figure legends**

Line 630 & 682 & 695 & 716 & 728 – think plural of asterisk is asterisks, not asterixis

Line 674 -5 – legend refers to blue and gray histograms but both are blue though different shades of blue

Figure 2D lacks a figure legend

Figure 6 legend could clarify A, B, and C as showing examples of shell positions. Clarify which part of legend referring to 6D.
