# Supplementary material for: PIWI silencing mechanism involving the retrotransposon nimbus orchestrates resistance to infection with Schistosoma mansoni in the snail vector, Biomphalaria glabrata
Source: PLoS Negl Trop Dis. 2021 Sep 8;15(9):e0009094. doi: 10.1371/journal.pntd.0009094 (PMC8462715; doi:10.1371/journal.pntd.0009094)
Supplement: Attachment — Submitted filename: Responses to reviewers comments.docx [file pntd.0009094.s005.docx]

Point -by-point responses to reviewers **PNTD-D-21-00019) - [EMID:23de7796b6f27ecd]**



**MK**

**Point-by-Point responses to reviewer's 1’s questions (answers are written in red font)**

We are thankful for the comments and suggestions that both reviewers made and have incorporated them into revising the manuscript. We hope the manuscript now reads better and is more straightforward version of the original submission. We hope that the changes we have made in revising the manuscript now meets with the requirement for publication in Plos NTDs.

Reviewer #1: Please see attached document. I selected accept with minor revisions as I do not think any additional experiments need to be carried out. However, I believe the manuscript should be comprehensively revised.
This manuscript describes the findings of a comprehensive study on the interplay of piwi and nimbus, and their potential role in the resistance or susceptibility of Biomphalaria snails to schistosome infection. This research is original and important to the field. It will also be of interest to researchers in other fields such as epigenetics and invertebrate immunology. Overall I recommend the following major editing.

We thank this reviewer for his/her kind sentiments about our study.

I recommend the following points be addressed and

I’ve listed suggested edits.
•       Overall, there was variation in formatting and writing style, perhaps as there are multiple authors. I recommend revising the paper so it is more uniform.
•     To limit the variation in formatting and writing styles we have limited the writing of this revised manuscript to only two of the authors to manage the flow and style of writing.

  It would be helpful to clarify why the 2 different siRNAs were used together rather than separately.

We did not intend to use the siRNAs together. They were mixed by serendipity. The graduate performing the experiment made a mistake. She did not realize the two siRNA stocks were to be used separately and combined them into a single tube with the PEI cationic carrier, so this was due to serendipity which has now been mentioned in the revised manuscript. However, because the protocol worked so well in silencing the expression of the PIWI encoding transcript, we continued to this protocol for all the biological replicates.

•       Can the authors provide any explanation for why in Fig 3, UNI siRNA treated unexposed BS-90 showed a much greater decrease in nimbus expression than in UNIsiRNA treated and schistosome-exposed BS-90?

Technically, this ia a non-specific result and we expected expression of nimbus to remain unchanged as in the control snails regardless of whether the snails were exposed or not to S. mansoni. Upregulation of nimbus RT occurs only in exposed susceptible snails, never in the resistant BS-90 snail unless, as this result shows, the transcript encoding piwi has been silenced before exposure. The downregulation of nimbus RT in the mock UNI siRNA transfected snails is similar to what is observed in control snail as was expected. The difference in intensity of down regulation is, therefore, non-specific and this point has now been clarified in the revised manuscript.

•        This was a comprehensive study comprising several different methods/experiments. A flow chart or graphic giving overview of all methods would be helpful to readers.
•     A flow chart has now been included (Fig. ) in the revised manuscript as suggested.

  Likewise, a graphic summarizing the results would be helpful.
•      The flow chart is comprehensive so might another summary be redundant?

The description of methods used to acquire sequences in order to design primers for qPCR were confusing (lines 209-223).

`Thanks for the suggestion. We have revised the writing of this section and hope it more clearly now for the reader.

There was mention of exploring the phylogeny of the B. glabrata orthologs but it isn’t clear why this was performed and I didn’t see the resulting data anywhere.
Thank you for the reviewer for pointing this out to us. This omission has now been corrected and the phylogeny data has now been included in the revised version of the manuscript.

       If space in journal is limited, Figures 2B and 2C do not appear to be absolutely necessary. A table with number cercariae shed might be more impactful.

These figures are now included in the revised manuscript as supplementary figures 1 and 2 since we no longer have the data for number of cercaria shed. The graduate student who did this experiment left for Saudi Arabia after graduation and is no longer in touch with the PI.

The significance of the use of the BPPA hTERT inhibitor, and that it stops cercarial shedding when snails treated with it 2 weeks post-infection, was not clear.

A better explanation has now been provided in the revised manuscript for why the BBPA RT inhibitor was also examined in the study. Although both lamivudine and BPPA are RT inhibitors they are expressed at different times since Lamivudine blocks infection in pre-treated snails and BPPA only after 2 weeks infection. Using Lamivudine after 2 weeks post infection does not completely block infection of the snail thus illustrating that the mechamism of involvement of the Piwi/nimbus interplay in snail susceptibility to *S. mansoni* infection kicks in at early and not late stages of infection.

•        It was not clear why the 2 siRNAs were used simultaneously not individually.

The reason for using 2siRNAs together has been provided above.

**MINOR COMMENTS- Reviewer 1**
Suggested edits
Line 37-38 – swap permissive and non-permissive
Corrected as suggested

Line 42 – join acetyl and transferase = acetyltransferase
Corrected as suggested

Line 42 – need to separate histone acetyl transferase and histone deacetylase with comma
Corrected as suggested

Line 139 – suggest changing “obtain leads” to “obtain candidates” to avoid having leads twice in same sentence
Corrected as suggested

Line 194 – insert comma after “(TmHMM)
Corrected as suggested

Line 203 – please include more detail on how specific transcripts to be examined vi two step qPCR were selected.

More details have been provided on how the transcripts were selected for two step PCR
Line 209 – not clear what “#7050” refers to. Should this citation be a number in refs rather than name and year?
This has been corrected

Line 216 - join acetyl and transferase = acetyltransferase, insert comma after (BgHAT)
Corrected as suggested

Line 228 – join “Bio” and “systems”. Is Wolston Warrington the correct address? I could not find

it on a map. Maybe just include Warrington
These have been corrected as suggested.

Lines 208-226 – there is  lack of consistency in wording of two step RT qPCR (i.e. sometimes two step included other times not), and whether given in full or abbreviated
These have been corrected as suggested.

Lines 244-75 – P of BgPiwi was uppercase previously
The formatting has been corrected and is now consistent throughout the manuscript

Line 246 – would change wording to “either dsRNA or siRNA, complexed with PEI”
Corrected as suggested

Line 274 – remove “in”
Corrected as suggested

Line 286 – have already given Sigma address in full (line 252)
Thanks for pointing this out. It’s been corrected as suggested.

Line 286 – insert comma after “MO)”
Corrected as suggested

Line 310 – should the comma be replaced with a semicolon?
Corrected as suggested

Line 317 & 328 – perhaps change “presented” to added
Corrected as suggested

Line 330-1 – would reword description of incubations at different temperatures, e.g., The Top Brite was set for 2 min at 37C, then 2 mins at 75C, and then 30 min at 37C
Reworded as suggested.

Line 352 – numbers can be in same box, don’t’ need separate box for each number
Corrected as suggested

Line 366 – De-novo doesn’t need uppercase d or a hyphen
Corrected as suggested

Line 368 – should be one sentence, no period after “temperatures”
Corrected as suggested

Line 395 – “demonstrates” rather than “demonstrate”
Corrected as suggested

Line 399, “he” should be “the”
Corrected as suggested

Line 426 – insert comma after “nimbus”
Corrected as suggested

Line 428 – after “PEI)” insert a comma and then “ the”
Corrected as suggested

Line 456 – remove “either”
Corrected as suggested

Line 509 – insert space after “insects”
Corrected as suggested

Line 514-19 – insert some commas in lists of genes, overall this sentence doesn’t read well
Corrected as suggested

Line 528 – insert commas in list of transcripts (e.g. after (HAT) and after (HDAC)
Corrected as suggested

Line 546 – replace “preformed” with “examined” or “recorded”
Corrected as suggested

Line 551 – delete “reveals the repertoire”
Corrected as suggested

Line 592 – can put 2 numbers in same [ ]
Corrected as suggested

Figure legends

Line 630 & 682 & 695 & 716 & 728  – think plural of asterisk is asterisks, not asterixis
Corrected as suggested

Line 674 -5 – legend refers to blue and gray histograms but both are blue though different shades of blue
Corrected as suggested

Figure 2D lacks a figure legend

Apologies for this omission. A figure legend has now been provided. This is now supplementary figure 2 in the revised manuscript.

Figure 6 legend could clarify A, B, and C as showing examples of shell positions. Clarify which part of legend referring to 6D.

This figure legend has now been re-written in the revised version of the manuscript.

**Reviewer #2:**

The manuscript entitled “PIWI silencing mechanism involving the retrotransposon nimbus orchestrates resistance to infection with Schistosoma mansoni in the snail vector, Biomphalaria glabrata” is focusing on to decipher the role of bgPIWI snail resistance to one of its parasite, S. mansoni. Firstly, the authors uses a comparative transcriptomic approach to analyze the differential expression genes between permissive and non per-issive BS90 snails, a model of choice to investigate the link between snail epigenetics and resistance. Then, by a classical QPCR approach , they confirmed the differential gene expression for BgPiwi, BgMTn BgCBx1, BgHAT et HDAC between BS90 (non permissive) and another snail strain BBO2 (or BB02?) which is susceptible to S. mansoni. siRNA experminents targeting bgPIWI render resistant BS90 snails susceptible. A correlation has been done between bgPIWI and a non LTR retrotransposable element nimbus expression. RT inhibition by lamivudine
affect snail susceptibility against S. mansoni.
This study could be of relevance for the field. However, I have major concerns regarding the method used and the interpretation of the results. These concerns are detailed below.

MAJOR COMMENTS
Statistics methods used could be improved. For instance, multiple time points (parasite challenge) could be compared globally using a Kruskal-Wallis or an ANOVA test (depending on normality and homoscedasticity of the distributions) and post-hoc test can be then performed to identify pairwise differences. No test on normality data was mentioned for all analyzed data. Please provide more details for the statistical analysis

The authors also do not report the validation of the primer couples they are using in terms of PCR efficiency; thus, it is not clear whether they meet the conditions required for DeltaDeltaCt use. Primers were designed for CDS of transcript and validated beforehand as we have done in all our publications utilizing qPCR, Ittiprasert et al 2009. Note there are also statistics that are specifically dedicated to qRT-PCR (Pfaffl method).

The data generated during this research were statistically analyzed by two-way analysis of variance (ANOVA). This is because the basis of our experimental approach was to examine the relationship between two independent variables i.e. snail susceptibility status (BS90 or BB02) and parasitic exposure time, and their combined effect on the dependent variable i.e. specific gene expression levels. This assessment would have been impossible using a one-way ANOVA which is a parametric equivalent of the Kruskal–Wallis test since the test is an appropriate statistical measurement of the effect of only one independent variable on a dependent variable. The variance of each of the data groups was determined using F-test. The results revealed that there was no statistically significant difference between the variances of the experimental groups, which denotes the homoscedastic distribution of the data sets. Thus, within each group, we performed multiple comparisons between the test results and the control (baseline) results.

In the abstract, the authors mentioned that comparative analysis will be performed on permissive and non-permissive BS90 snails, and the strain BBO2 was not indicated . A

ll in all, the reader might reasonably expect that all comparisons are made between permissive and not permissive BS90, but this is not the case. Why ?  We have clarified our rationale for using BBO2 and BS-90 in the revised manuscript.

To my mind, it will reinforce the conclusions of the paper and it will be a best straight strategy to claim on the bgPiwi role.  All results performed with the BBO2 strain could be extrapolated for the BS90 F2 (permissive condition) ?

We agree with the reviewer but using the representative resistant BS-90 and susceptible BBO2 snail gave us the confidence that these transcripts identified initially with the RNA -seq of the plastic phenotype in BS90 was relevant in snail anti-schistosome mechanism(s) if validated in actual -well characterized resistant and susceptible snails.

It will be elegant to reinforce the conclusion to have the results from qPCR analysis of RNA from permissive BS90 exposed to parasite. In the same way, treatment with inhibitors could be performed on permissive BS90 snails and compared to non-permissive snails.

Please for all QPCR analysis, give the same scale for the y-axis. For example in Fig2A,  bgpiwi expression seems to be unchanged  with 200 ng/ml of inhibitor whereas its expression is decreased compared to the untreated snails. Intersection at x-axis, the y co-ordinate should be 1

This has now been corrected as suggested.

Please indicate the prevalence for the snails treated and not treated with inhibitors. Prevalence of infection? We examined if snails were either shedding or not and scored them as being patent for *S. mansoni* if they released cercariae.

According to me, Figure 2C ca be added in supplementary data.

Note that no legend is associated with the figure 2D This was noted and is now a supplementary figure as suggested by the reviewer. A figure legend has also been provided in the revised manuscript.

In the manuscript, the authors report that “Treatment of the snails with 464 lamivudine at day 14 post-exposure, however, showed some snails shed cercariae when 465 the drug was utilized later after infection.” Please indicate for how many snails (%) , this observation has been done. Also, if I understand well, this treatment can affect parasite development.

All the snails treated late with either 100ng/ml or 200ng/ml of Lamivudine released cercariae whereas none (100%) of the snails treated with this drug before exposure were found to release parasites. Looking at parasite development within the snail will require histology to determine the presence of encapsulated parasites and this was beyond the scope of the present study.

So, in Fig5A, what is the developmental stage of the parasites in the BBO2 snails exposed to lamivudine ? Are they killed, encapsulated or present as sporosysts but unable to grow. Please provide further discussion /clarification.

Examination of the effect of lamivudine on the developing intramolluscan larval stages was beyond the scope of the present study. We are currently examining the effect of this inhibitor on in vitro sporocysts developing in co-culture tissue culture with BGE cells.

Reviewer 2
L2 : 25°C (permissive) maybe non permissive ?

This has been corrected as suggested
Please harmonize writing for PIWI, Piwi or piwi

There is now consistency in writing PIWI

Please indicate the origin of BS90 (BS-90 ?) as done for the other snail strains.
This has been modified accordingly to the correct format, BS-90

L193 : Pfam

Corrected as suggested

L196 : clarify different categories
corrected as suggested

L188 : 2 hours ?

L209 : Format the reference

Corrected as suggested

L271 Edit a space between TR and nimbus
corrected as suggested

L376 and 396 : hours ?

Corrected as suggested

Addressing these points may require minor revisions and supplementary experiments, but will significantly improve the quality of the manuscript, which has great potential to provide novel insight into Biomphalaria - Schistosoma complex interaction

--------------------

PLOS authors have the option to publish the peer review history of their article (<a href="<https://journals.plos.org/plosntds/s/editorial-and-peer-review-process#loc-peer-review-history>" target="_blank">what does this mean?</a>). If published, this will include your full peer review and any attached files.<br><br>

If you choose “no”, your identity will remain anonymous but your review may still be made public.<br><br>

<b>Do you want your identity to be public for this peer review?</b> For information about this choice, including consent withdrawal, please see our <a href="<https://www.plos.org/privacy-policy>" target="_blank">Privacy Policy</a>.

Reviewer #1: No

Reviewer #2: No


Figure Files:
While revising your submission, please upload your figure files to the Preflight Analysis and Conversion Engine (PACE) digital diagnostic tool, [https://pacev2.apexcovantage.com](https://pacev2.apexcovantage.com/). PACE helps ensure that figures meet PLOS requirements. To use PACE, you must first register as a user. Then, login and navigate to the UPLOAD tab, where you will find detailed instructions on how to use the tool. If you encounter any issues or have any questions when using PACE, please email us at figures@plos.org.
 
Data Requirements:
Please note that, as a condition of publication, PLOS' data policy requires that you make available all data used to draw the conclusions outlined in your manuscript. Data must be deposited in an appropriate repository, included within the body of the manuscript, or uploaded as supporting information. This includes all numerical values that were used to generate graphs, histograms etc.. For an example see here: <http://www.plosbiology.org/article/info%3Adoi%2F10.1371%2Fjournal.pbio.1001908#s5>.
 
Reproducibility:
To enhance the reproducibility of your results, PLOS recommends that you deposit laboratory protocols in protocols.io, where a protocol can be assigned its own identifier (DOI) such that it can be cited independently in the future. For instructions see <https://journals.plos.org/plosntds/s/submission-guidelines#loc-methods>


***This is an automated email***

__________________________________________________
In compliance with data protection regulations, you may request that we remove your personal registration details at any time.  (Use the following URL: <https://www.editorialmanager.com/PNTD/login.asp?a=r>). Please contact the publication office if you have any questions.

Point -by-point responses to the reviewers

**Reviewer 1**

**Reviewer 2**

The data generated during this research were statistically analyzed by two-way analysis of variance (ANOVA). This is because the basis of our experimental approach was to examine the relationship between two independent variables i.e. snail susceptibility status (BS90 or BB02) and parasitic exposure time, and their combined effect on the dependent variable i.e. specific gene expression levels. This assessment would have been impossible using a one-way ANOVA which is a parametric equivalent of the Kruskal–Wallis test since the test is an appropriate statistical measurement of the effect of only one independent variable on a dependent variable. The variance of each of the data groups was determined using F-test. The results revealed that there was no statistically significant difference between the variances of the experimental groups, which denotes the homoscedastic distribution of the data sets. Thus, within each group, we performed multiple comparisons between the test results and the control (baseline) results.
