# Supplementary material for: PIWI silencing mechanism involving the retrotransposon nimbus orchestrates resistance to infection with Schistosoma mansoni in the snail vector, Biomphalaria glabrata
Source: PLoS Negl Trop Dis. 2021 Sep 8;15(9):e0009094. doi: 10.1371/journal.pntd.0009094 (PMC8462715; doi:10.1371/journal.pntd.0009094)
Supplement: Attachment — Submitted filename: R2 point-by-point responses to the reviewers.docx [file pntd.0009094.s006.docx]

**Point-by-point responses to comments made by reviewers.**

The lines where corrections have been made in the R2 version of the manuscript are shown in blue font. Comments by reviewer #1 that were unclear are highlighted in yellow

**Reviewer #1**

**line 44 (line 44)- differential expression doesn't need uppercase letters (Differential Expression)**

Corrected as suggested

**line 83 (line 86) - "became" should be "become"**
Corrected as suggested

**line 109 (line 113) - check if unnecessary space between "long-" and "term"
Corrected as suggested**

**line 155 (line 161)- insert "temperatures" after (25C)**
Corrected as suggested

**lines 166-168 (lines 171-172) - could these 2 sentences be merged, in 1st sentence state that BS-90 resistant to S. mansoni NMRI strain, and in 2nd sentence state that BS-90 resistant to S. mansoni strains from Old World and New World.**
The sentence has been re-written as suggested by the reviewer

**line 177 – (line 181) should "exposure" be plural?**
Corrected as suggested

**line 180 (line 184)- remove space in bri address**
Corrected as suggested

**line 211 (lines 221-222)- transcriptome should be plural as multiple transcriptomes generated**
Corrected as suggested

**line 214 (line 225) - insert comma after "(32C) corrected as suggested**

Corrected as suggested

**line 223 – (line 235) Table 1 lacks a title and not clear if upregulated and downregulated refers to in 32C or in 25C. Legend for table would be helpful.**

Corrected as suggested. A more informative and descriptive title has now been provided.

**line 228 (line 244) - why is the formatting of this citation not a number?**

This mistake has been corrected.

**line 235 (line 252) - check if unnecessary space between "RNA-" and "seq"**
corrected as suggested

**line 237 (line 254) - insert "temperatures" after "(25C)"**
corrected as suggested

**line 267 (line 258) - give BLAST in full earlier (line 234)**
corrected as suggested

**line 289 (line 311) - check for unnecessary spaces within equation in subscripts**
corrected as suggested

**line 311 (line 337) - not clear which reagent "(Sigma)" refers to**

corrected as suggested

line 314 - don't need "("  as have one line 313

corrected, missing bracket was inserted **line 340**

**line 318 (line 344) - don't need uppercase T for "Total"; don't think it is necessary to include "(by serendipity**)"
corrected as suggested

**line 329 (line 355)- please correct "in at"**

corrected as suggested

**line 349 (line 378) - please check if need space in siRNABgPIWI**
corrected as suggested

**line 383 (Line 413)- suggest changing verb from "was presented to a coverslip" to "was applied to a coverslip"**
corrected as suggested

**line 427 (line 458)- unnecessary space after "mansoni"**
corrected as suggested

line 450 - "No" should be "No." (unclear)

line 451 - unnecessary space after "set" (unclear)

**line 454 and 455 (line 489)- check if space missing between "1" and "and"**

corrected as suggested

**line 468 (line 504) - title for table not accurate as table not showing multiple alignments, it shows the degree of identity between homologs**

corrected as suggested

**line 471 – (line 557) suggest replacing "however" with "furthermore**"
corrected as suggested

**line 482 – (line 598) suggest changing "infection" to exposure as BS-90 maintained at 25C**

corrected as suggested

**line 497 (lines 535, 553)- insert commas after "transfected" and "normal snails". Also suggest not using the term "normal". Instead suggest using "untransfected". Same for line 509 regarding use of "normal".**
corrected as suggested

**line 518 and following - lack of consistency in BgPiwi, p often lowercase in remainder of manuscript (e.g., lines 525, 526, 533, 534) lines 565, 566,573,574,587,588**

corrected as suggested to upper case format as follows: *BgPiwi*

**lines 553 & 554 (line 599)- suggest using S. mansoni miracidia/sporocysts instead of "parasite" for sake of clarity and consistency**

Corrected as suggested

**line 612 –(line 673)insert comma after "(BgHAT)"**

corrected as suggested

**line 679 – (741) remove space after "54" and insert before "55"**
corrected as suggested

**line 684 (line 755) - suggest changing wording to clarify that referring to exposure of resistant and infection of susceptible, rather than referring to both using "infection"**

corrected as suggested

**line 681 – (lines 680, 743) check for consistency in how write piwi when referring to gene**
corrected as suggested

**line 686 – (line 756) suggest changing miracidium to plural**

**line 687 – (line 752) do the authors mean systematic rather than systemic?**

Reviewer #2: Accept

--------------------

<b>Summary and General Comments</b></br></br>
Use  this section to provide overall comments, discuss strengths/weaknesses of the study, novelty, significance, general execution and scholarship. You may also include additional comments for the author, including concerns about dual publication, research ethics, or publication ethics. If requesting major revision, please articulate the new experiments that are needed.

Reviewer #1: This manuscript describes research that was done to  increase our understanding of the complex molecular interactions between B. glabrata snails and Schistosoma mansoni. It is of interest to researchers of this host-parasite system, as well as to those in the fields of host-parasite interactions, molecular parasitology, and vector biology, to name a few. The research is very novel, interesting, and significant to the fields mentioned above.
I have suggested multiple edits in a separate section as well as a few concerns or suggestions below.

lines 247-250 - (lines 516 to 524) It seems that if qPCR carried out to verify RNA-seq data from BS-90 maintained at 25C and 32C, then qPCR should also be carried out on BS-90 maintained at 32C. It is valuable to carry out qPCR on susceptible BBO2 but i think it should also be carried out on BS-90 maintained at 32C seeing as original RNA-seq data from BS-90 maintained at 32C. Lines 420-430 mention carrying out qPCR on BS-90 maintained at 32C but earlier in manuscript, and in text following from lines 430 it is not clear when BS-90 maintained at 32C used for qPCR.

RNA from BS-90 snails cultured at 32^o^C were indeed utilized in parallel for qPCR (Fig. 3A). The sentence has been re-written for clarity in lines 516 to 524 in the revised manuscript.

**Line 457 – (line 504) BgPiwi described as being highly conserved by amino acid identity around 40% which isn't that high. Would just state that it is conserved.**

This has been corrected as suggested

Reviewer #2: Authors have answered point by point to the several questions that i have raised.
The manuscript can be published

--------------------

PLOS authors have the option to publish the peer review history of their article (<a href="<https://journals.plos.org/plosntds/s/editorial-and-peer-review-process#loc-peer-review-history>" target="_blank">what does this mean?</a>). If published, this will include your full peer review and any attached files.<br><br>

If you choose “no”, your identity will remain anonymous but your review may still be made public.<br><br>

<b>Do you want your identity to be public for this peer review?</b> For information about this choice, including consent withdrawal, please see our <a href="<https://www.plos.org/privacy-policy>" target="_blank">Privacy Policy</a>.

Reviewer #1: No

Reviewer #2: No
